# Surgical repair of severely incompetent quadricuspid truncal valve

**DOI:** 10.1093/jscr/rjab427

**Published:** 2021-09-29

**Authors:** Rodrigo Sandoval Boburg, Migdat Mustafi, Michael Hofbeck, Christian Schlensak

**Affiliations:** Department of Thoracic and Cardiovascular Surgery, Tübingen University Hospital, 72076 Tübingen, Germany; Department of Thoracic and Cardiovascular Surgery, Tübingen University Hospital, 72076 Tübingen, Germany; Department of Pediatric Cardiology and Intensive Medicine, Tübingen University Hospital, 72076 Tübingen, Germany; Department of Thoracic and Cardiovascular Surgery, Tübingen University Hospital, 72076 Tübingen, Germany

## Abstract

The surgical management of truncus arteriosus poses a constant challenge for the cardiac team treating the patient. A correct diagnosis, surgical therapy and post-operative management are crucial for the survival of the patient. Almost 30% of the patients show an abnormal number of leaflets in the truncal valve (TV), the majority being quadricuspid valves. Additionally, around 25% of the patients show some degree of TV incompetence. We demonstrate an effective way to reconstruct incompetent, quadricuspid valves with good post-operative outcome.

## INTRODUCTION

Common arterial trunk (TAC) is a rare complex congenital heart defect with an incidence of 6–10/1 00 000 live births [1]. Children born with TAC require surgery at a very young age. Operative outcome in neonates is good and it is recommended to perform surgical correction before heart failure, or any organ insufficiency develops [2, 3]. One of the most common challenges that may arise when performing surgery on these patients is the repair of an abnormal and severely regurgitant truncal valve (TV), which occurs in ~25% of patients with TAC [4]. There have been reports of TV having two to six leaflets [5, 6]. Among the abnormal TV, the quadricuspid TV is the most common one [5]. If the valve is severely regurgitant repair cannot be avoided when performing surgical correction. Tricuspidalization of the valve is a reproducible and safe alternative when treating this condition with good outcomes [7, 8]. Here, we explain step-by-step, a simple way to perform this procedure.

## CASE

**Figure 1:**
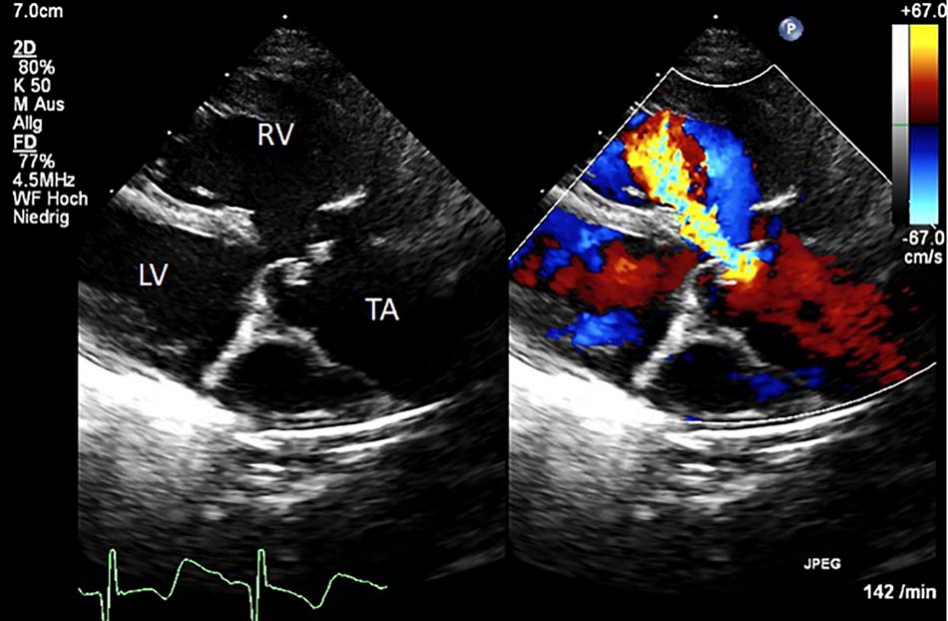
Color Doppler echocardiography in the parasternal long axis view shows severe TV regurgitation. RV: right ventricle, LV: left ventricle, TA: truncus arteriosus.

We present a case of a female patient referred at 10 days of age with 22q11.2 deletion syndrome, TAC type A2 of the van Praagh classification with a regurgitant, quadricuspid TV and VSD (Fig. 1). The patient had signs of heart failure and respiratory insufficiency and had to be intubated and transferred to the pediatric intensive care unit pre-operatively. After hemodynamic stabilization, surgery was performed.

**Figure 2:**
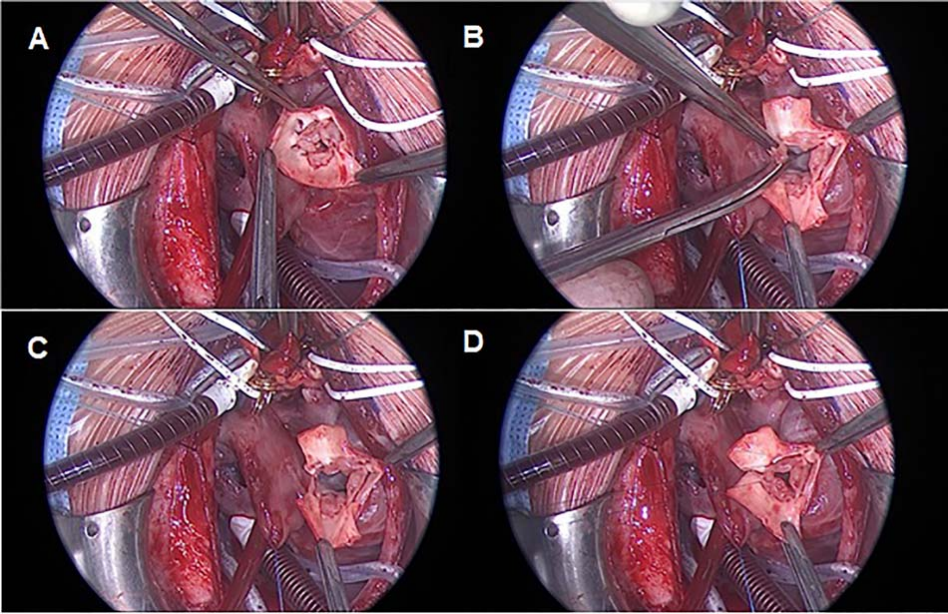
TV reconstruction. (**A**) Leaflet identification. (**B** and **C**) Leaflet resection. (**D**) Local annuloplasty and aortic valve suture.

Median sternotomy is performed, and anatomy is confirmed. The pulmonary arteries are mobilized as much as possible. To achieve cardiopulmonary bypass (CPB), the arterial cannula is inserted in the aortic arch, venous cannulas in the superior and inferior venae cavae. After starting CPB and induction of ventricular fibrillation, the right atrium is opened and cold cardioplegic solution is infused into the coronary sinus under pressure control (Buckberg’s solution, 30-ml/kg/body weight induction, pressure < 30 mmHg). This is followed by the transverse opening of the trunk vessel with a distance to the branches of the coronary vessels. Inspection of the truncus valve, in our case it was severely incompetent in the pre-operative transesophageal echocardiography, showed four cusps and asymmetrical sinuses. Three sinuses and the associated valve leaflets are roughly the same size; the fourth sinus and corresponding valve leaflet are much smaller and, most importantly, have no relation to a coronary ostium. The hypoplastic valve leaflet is resected together with the sinus of the trunk vessel up to the commissures of the adjacent valve cusps (Fig. 2).

These adjacent commissures are then sutured longitudinally starting from the valvular ring with a continuous 7–0 polypropylene suture.

Care must be taken that through the resection of the cusp and the corresponding sinus, no valvular stenosis is caused. In our case, the ring diameter was −1 Z score after reconstruction. The further course of the operation is carried out as standard with resection of the pulmonary vessels from the trunk and connection to the RVOT by means of an REV maneuver.

Weaning of CPB and chest closure is performed in a usual manner.

**Figure 3:**
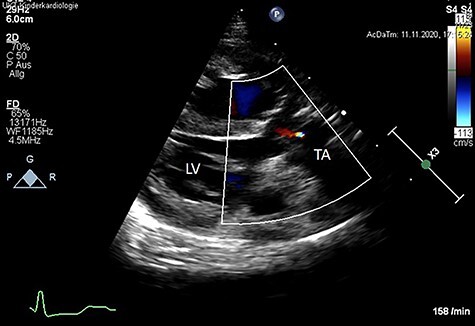
Post-operative color Doppler echocardiography (parasternal long axis view) reveals a competent TV with just a minor trace of central regurgitation. Ao: Aorta, LV: left ventricle, TA: truncus arteriosus.

Following surgery, the patient showed a good result and satisfactory recovery. The patient was discharged from the hospital at the third post-operative week. Post-operative echocardiography revealed a good result without TV stenosis or regurgitation (Fig. 3).

## DISCUSSION

In most cases, the TV incompetence is mild and requires no surgical therapy at the time of TAC correction. If the TV incompetence is moderate or severe, correction is necessary. A TV replacement with a mechanical prosthesis or a homograft, although technically feasible because of the usually large valvular ring, is associated with a higher morbidity and mortality due to requirement of life-long anticoagulation and/or re-operations.

An alternative to replacement is TV reconstruction; this option is more challenging and not performed as often. Naimo *et al.* [8] reported 14 patients who underwent TV surgery with different techniques at the time of TAC repair. However, the surgical techniques in this study are mentioned but not explained in detail. We describe a detailed surgical technique which is reproducible in patients with a quadricuspid TV. In these cases, usually one of the sinuses is hypoplastic; we recommend the resection of the smallest sinus without coronary ostium. With this approach, a larger coaptation area of the remaining sinuses can be achieved. To avoid post-operative obstruction of the TV, it is important to ensure that after resection, the TV ring should not be smaller than −1 Z Score. The limitation of this study is that it has been performed in a single patient and therefore requires confirmation in a larger cohort of patients.

## Supplementary Material

TAC-shortened_version_(2)_rjab427
